# Different Mechanisms of Regulation of the Warburg Effect in Lymphoblastoid and Burkitt Lymphoma Cells

**DOI:** 10.1371/journal.pone.0136142

**Published:** 2015-08-27

**Authors:** Muhammad Mushtaq, Suhas Darekar, George Klein, Elena Kashuba

**Affiliations:** 1 Department of Microbiology, Tumor and Cell Biology (MTC), Karolinska Institute, Stockholm, Sweden; 2 R. E. Kavetsky Institute of Experimental Pathology, Oncology and Radiobiology, NASU, Kyiv, Ukraine; IRCCS National Cancer Institute, ITALY

## Abstract

**Background:**

The Warburg effect is one of the hallmarks of cancer and rapidly proliferating cells. It is known that the hypoxia-inducible factor 1-alpha (HIF1A) and MYC proteins cooperatively regulate expression of the *HK2* and *PDK1* genes, respectively, in the Burkitt lymphoma (BL) cell line P493-6, carrying an inducible *MYC* gene repression system. However, the mechanism of aerobic glycolysis in BL cells has not yet been fully understood.

**Methods and Findings:**

Western blot analysis showed that the HIF1A protein was highly expressed in Epstein–Barr virus (EBV)-positive BL cell lines. Using biochemical assays and quantitative PCR (Q-PCR), we found that—unlike in lymphoblastoid cell lines (LCLs)—the MYC protein was the master regulator of the Warburg effect in these BL cell lines. Inhibition of the transactivation ability of MYC had no influence on aerobic glycolysis in LCLs, but it led to decreased expression of MYC-dependent genes and lactate dehydrogenase A (LDHA) activity in BL cells.

**Conclusions:**

Our data suggest that aerobic glycolysis, or the Warburg effect, in BL cells is regulated by MYC expressed at high levels, whereas in LCLs, HIF1A is responsible for this phenomenon.

## Introduction

Burkitt lymphoma (BL) is a B-cell derived childhood malignancy that is endemic in the rain forest areas of tropical Africa [1]. Almost all cases of endemic BL are associated with Epstein–Barr virus (EBV) infection. The main characteristic of both EBV-positive and-negative cases of BL is an increased production of the MYC oncoprotein, caused by chromosomal rearrangements [2]. Chromosomal translocation in BL cells always juxtaposes the MYC-encoding gene (NM_002467) to an immunoglobulin enhancer element (IgEE) [3, 4]. As IgEEs are specifically active in mature B cells, their translocation to *MYC* results in inappropriately high expression levels of MYC, which gives cells proliferative capacity regardless of EBV infection. BL cells show the ability to proliferate in soft agar and can produce tumors in experimental animals, i.e. SCID [5] and NUDE [6] mice. Moreover, MYC activates the transcription of genes that are involved in glycolysis [7]. It is well known that tumor and rapidly proliferating cells are distinguished from normal cells by a difference in glucose metabolism. In normal physiological conditions, oxidative glycolysis takes place when one glucose molecule is converted into two pyruvate molecules. Subsequent oxidation of pyruvate to CO_2_ produces about 36 molecules of ATP per molecule of glucose [8]. At a lower concentration of oxygen, anaerobic glycolysis is activated, and the cells convert most of pyruvate to lactate that is secreted by the cells. As a result, only 2–4 molecules of ATP are produced, compared with pyruvate oxidation [9]. Tumor and rapidly proliferating cells convert pyruvate to lactate along with its oxidation under normoxic conditions: in other words, cells show the Warburg effect.

We have shown earlier that lymphoblastoid cell lines (LCLs) can also exhibit a Warburg effect [10], as do malignant cells. The major driver of this “aerobic” glycolysis regulation in LCLs is the stabilization of hypoxia-induced factor 1 alpha (HIF1A, NP_001521), caused by inactivation of prolylhydroxylases 1 and 2 (PHD1, NP_542770 and PHD2, NP_071334, respectively) by binding to EBV-encoded nuclear antigens (EBNA-5 and EBNA-3) [10].

However, not just HIF1A is involved in regulating the expression of a set of genes involved in glucose metabolism. Many genes of this pathway are also direct targets of MYC [9], [11], [12]. For example, both the transcription factors MYC and HIF1A can transactivate genes such as those encoding the glucose transporter (*GLUT*), hexokinase (*HK*), monocarboxylate transporter (*MCT*), pyruvate dehydrogenase kinase (*PDK*), phosphofructokinase (*PFK*), phosphoglycerate kinase (*PGK*), pyruvate kinase (*PK*), and lactate dehydrogenase A (*LDHA*). All these genes encode important enzymes of aerobic glycolysis [13], [14]. A loss of MYC’s function in a BL cell model with conditional *MYC* overexpression results in decreased expression levels of genes involved in glucose metabolism [12]. However, the mechanism of aerobic glycolysis in BL cells is not fully understood. Here we report that the MYC protein is the master regulator of the Warburg effect in BL cells, in contrast with LCLs. Inhibition of the transactivation ability of MYC had no influence on aerobic glycolysis in LCLs; in contrast, in BL cells it led to decreased expression of MYC-dependent genes and impaired LDHA activity.

## Material and Methods

### Cell culture

The EBV negative BL cell lines (Akata, BL28, BL41, BJAB, DG75, Mutu (clones 9 and 30), Oma clone 4, and Ramos), latency I EBV positive BL cell lines (Akata (+), BL28/95A, BJIAB/B95.8, Jijoye M13, Mutu I (clones 59 and 148), Oma clone 6, and Rael), EBV positive latency III BL cell lines (Akuba, BL16, BL18, BL41/95, Mutu III (clones 99 and 176), and RAJI), the established LCLs (051128—2 months old, 121028—5 months old, 111210 and 120214—8 months old), and a sub-line of BJAB that expressed EBNA-1 constitutively (see [15–17] for BL cell line description) were cultured at 37°C in Iscove's medium that contained 10% fetal bovine serum and appropriate antibiotics (see S1 Table). LCLs were established in our lab by infection of peripheral B-cells with the laboratory B95.8 strain of EBV. Peripheral B-cells were isolated from buffy coat (Karolinska Hospital, Stockholm) on Lymphoprep gradients and by two subsequent rounds of E-rosetting removed the T-cells. No permission from an ethical committee for B-cell isolation from buffy coat is needed.

In order to inhibit the binding between HIF1A and aryl hydrocarbon receptor nuclear translocator (ARNT or HIF1B, NP_001659), cells were cultured at 37°C in media that contained 5μM of Acriflavine hydrochloride (3,6-Diamino-10-methylacridinium chloride hydrochloride, Euflavine) (Sigma-Aldrich, St. Louis, MO, USA). Cells were harvested after 3 hours of treatment, to perform quantitative PCR (Q-PCR) and biochemical assays. To monitor cell proliferation, cells were counted at 3, 6, 20, 24, 32 and 56 hours after the beginning of treatment.

To prevent nuclear translocation of HIF1A protein, cells were treated with 5μM of 2-methoxyestradiol (2-MeOE2, Sigma-Aldrich). Cells were harvested after 3 hours of treatment, to perform quantitative PCR (Q-PCR).

To achieve the inhibition of a complex formation between MYC and MAX proteins, cells were treated with 100 μM solutions of 10058-F4 (5-[(4-Ethylphenyl)methylene]-2-thioxo-4-thiazolidinone) (Santa Cruz Biotechnology, Santa Cruz, CA, USA) and 10074-G5 (Sigma-Aldrich). Cells were harvested after 4 hours of treatment to carry Q-PCR out; treatment was prolonged for 16 hours for biochemical assays.

### Quantitative PCR (Q-PCR)

Total RNA was purified from cells using the RNA Purification Kit (Fermentas, Hanover, MD, USA). 1 μg of total RNA was taken for the cDNA synthesis, using a First Strand cDNA Synthesis Kit (Fermentas). The total reaction volume was 20 μl and the primer concentration was adjusted to a final concentration of 0.3 μM for Q-PCR. Reactions were performed, using SYBR Green/ROX Master Mix on a 7900 machine (Applied Biosystems, Foster City, CA). Primers for HIF1A1-responsive genes were following: *GLUT1* (NM_006516)—forward 5’-AAGGTGATCGAGGAGTT CTACA-3’, reverse 5’-ATGCCCCCAACAGAAAAGATG-3’; hexokinase-2 (*HK-2*) (NM_000189), forward 5’-GAGCCACCACTCACCCTACT-3’, reverse 5’-ACCCAAAG CACACGGAAGTT-3’; *LDHA1* (NM_005566) forward 5’-CTCCAAGCTGGTCATTATC ACG-3’, reverse 5’-AGTTCGGGCTGTATTTTACAACA-3’; *PDK1* (NM_002610) forward 5’-TCCTGGACTTCGGATCAGTGA-3’, reverse 5’-CGGATGGTGTCCTGAGA AGATT-3’; *PGK1* (NM_000291) forward 5’-TTAAAGGGAAGCGGGTCGTTA-3’, reverse 5’-TCCATTGTCCAAGCAGAATTTGA-3’; *MCT4* (NM_004207) forward 5’-T GTGTGCGTGAACCGCTTT-3’, reverse 5’-AAACCCAACCCCGTGATGAC-3’. As an internal control for standardization, a gene encoding TATA-binding protein (*TBP*, NM_003194) was used with the following primers: forward 5’-TTTCTTGCCAGTCTG GAC-3’, reverse 5’-CACGAACCACGGCACTGATT-3’. The PCR cycling conditions were 10 min at 95°C, 40 cycles of 10 s at 95°C and 1 min at 60°C. Applied Biosystems 7900 systems software was used for analysis.

Ct values were determined for the internal control (*TBP*) and for the test genes at the same threshold level in the exponential phase of the PCR curves.

Relative quantification (comparative Ct (DDCt) method) was used to compare the expression level of the test genes with the internal control. Three or four reactions (each in triplicate) were run for each gene and the standard deviation was calculated.

### Biochemical assays

BL cells and LCLs were assayed for the concentration of L-lactate and pyruvate, and for lactate dehydrogenase catalytic activity.

Colorimetric assays were performed as described by Bioassay Systems (Hayward, CA, USA) to assess the concentrations of medium lactate (L-Lactate Assay Kit, ECLC-100) and pyruvate (Pyruvate Assay Kit, EPYR-100), and also lactate dehydrogenase activity (Lactate Dehydrogenase Kit, DLDH-100). Briefly, to measure the lactate concentration, the cell culture medium was collected (when 2×10^6^ cells were seeded a day before). The absorbance was measured at 565 nm in comparison with the control reaction (with out enzyme) after incubation of reaction for 20 min at the room temperature. IMDM medium with out serum was used as a standard to exclude the influence of phenolsulfonphthalein (Phenol red). For pyruvate and lactate dehydrogenase assays, 3×10^6^ cells were sonicated in 100 μl of 100 mM potassium phosphate/2mM EDTA (pH 7.0). The absorbance of standards and unknowns was measured on a microplate reader at 570 nm.

### Western blotting

Total cell lysates were prepared, using the NP40 lysis buffer (1% NP40, 150 mM NaCl, 50 mM Tris, pH = 8) with a protease inhibitor cocktail (Roche AB, Stockholm, Sweden) and bovine serum albumin, BSA (0.5% w/v) as a nonspecific competitor. After SDS–PAGE, proteins were transferred to nitrocellulose and probed with mouse antibodies against HIF1A (Life Technologies, Carlsbad, CA, USA), and actin (Sigma-Aldrich). Secondary antibodies (anti-mouse IgG HRP conjugated) were from GE Healthcare Bio-Sciences AB, Uppsala, Sweden.

### Statistical analysis

GraphPad Prism software (version 6, GraphPad Software, La Jolla, CA, USA) was used to determine the means of the HIF1A expression (as a normalized ratio of HIF1A to actin signals) in the studied cell lines. Further analysis was performed on the combined mean of each set of experiments.

## Results

### HIF1A is expressed at high levels in LCLs and Latency III BL cells

**Fig 1:**
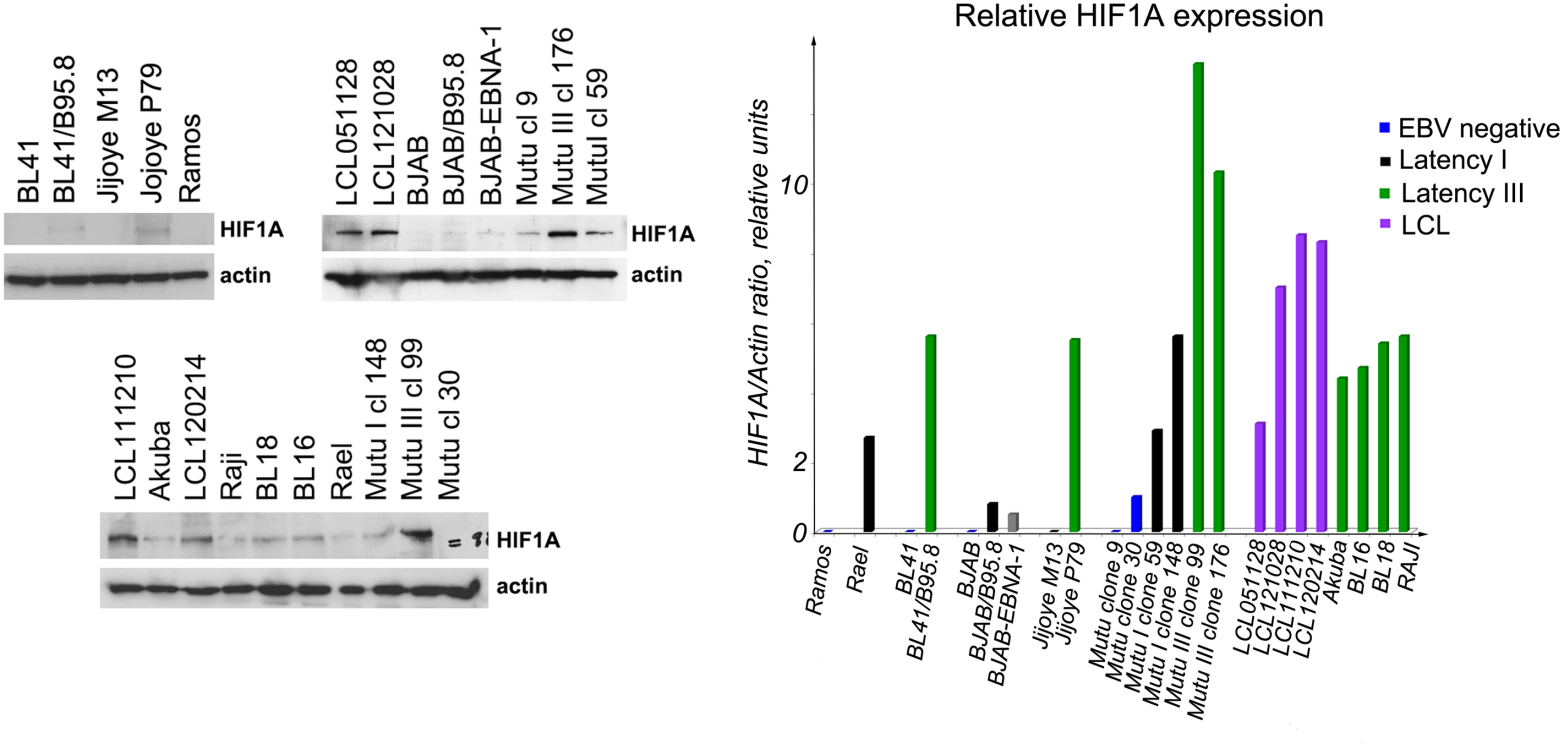
(A)—Expression levels of HIF1A protein assessed by Western blotting. Membranes were probed with mouse monoclonal antibodies against HIF1A and actin. HIF1A was highly expressed in EBV-positive cell lines with Latency III (LCLs, BL41/B95.8, Mutu III clones 99 & 176, Akuba, BL16, BL18, RAJI) compared with EBV-negative (Ramos, BL41, Mutu clones 9 & 30) and EBV-positive Latency I (Rael, BJAB/B95.8, Mutu I clones 59 & 148) cells; (B)—Relative HIF1A protein expression levels, normalized to the actin signal. The ratio of HIF1A to actin signals was calculated using the Western blotting results (Fig 1A). Note the higher expression of HIF1A in EBV-positive cells.

**Fig 2:**
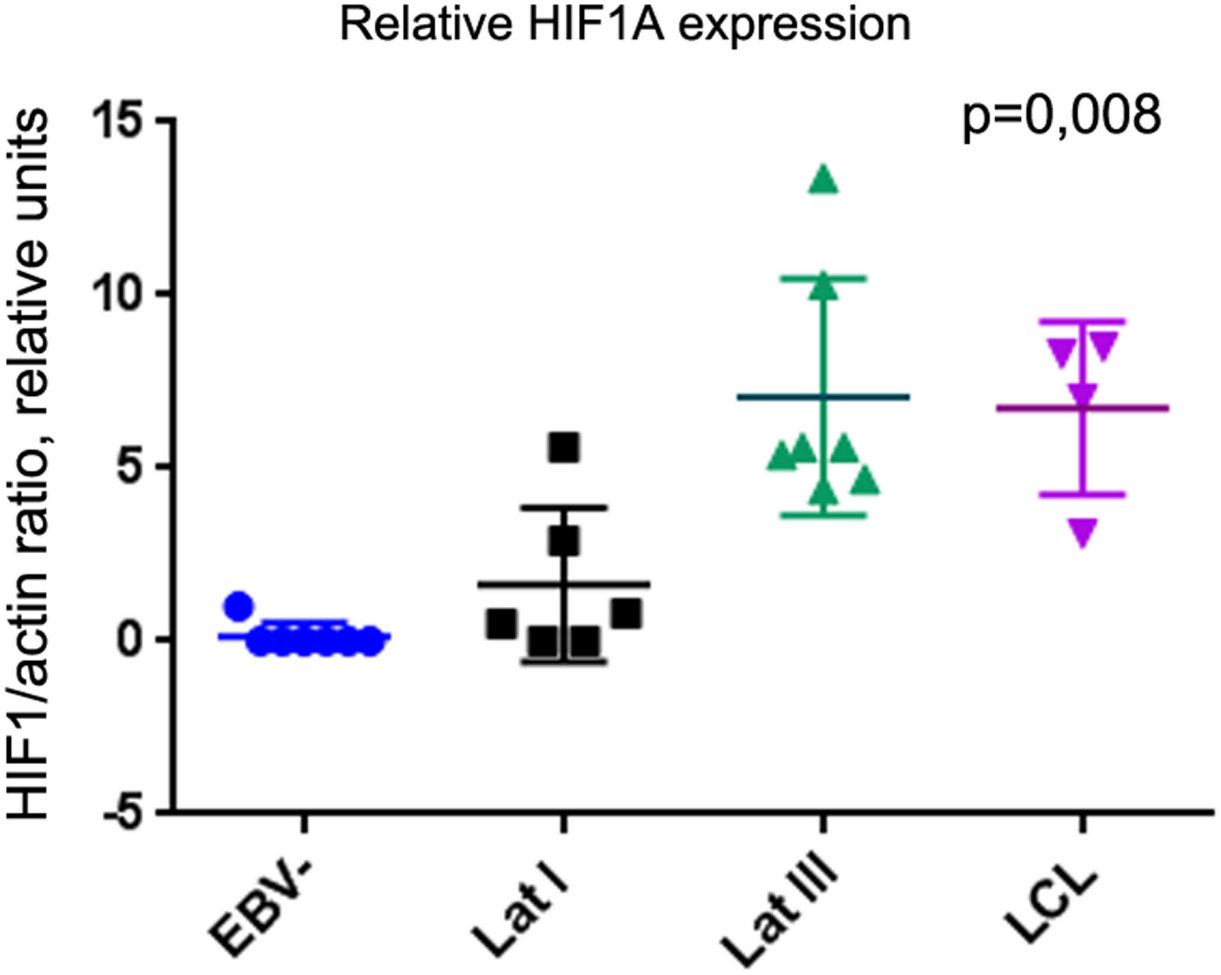
Statistical analysis of relative HF1A protein expression. Kruskal–Wallis tests were applied to four groups, comprising seven EBV-negative, six Latency I, seven Latency III BL cell lines, and four LCLs. HIF1A was expressed significantly higher in Latency III cells (p = 0.008<0.05).

Western blot analysis was performed to assess the expression of HIF1A at a protein level, using a monoclonal antibody against HIF1A (Fig 1). HIF1A was expressed at high levels in LCLs and Latency III BL cells, compared with the EBV-negative BL and EBV-positive Latency I BLs (Fig 1). For example, in Mutu III cells (clones, cls, 99 and 176) expressing the Latency III program, HIF1A levels were significantly higher than in EBV-negative Mutu cells (cls 9 and 30), or in Latency I convertant Mutu I cells (cls 59 and 148). In all, 24 cell lines were studied, among them four LCLs that were established 2–8 months prior to these experiments. A statistical analysis of the normalized HIF1A/actin ratio (Kruskal–Wallis statistics on four groups) showed a significant difference between EBV-negative BLs and Latency III cells (p<0.05). The statistical analysis of HIF1A expression in BLs and LCLs is shown in Fig 2. Presumably, the HIF1A was stabilized in LCLs and Latency III BLs at a protein level by inhibition of hydroxylation and subsequent degradation by proteasomes, consistent with a previous report [10].

We have shown earlier [10] that HIF1A plays a leading role in transactivation of many genes that are involved in aerobic glycolysis: thus, HIF1A was responsible for the Warburg effect in LCLs. Could HIF1A be responsible for aerobic glycolysis in BL cells?

### Genes encoding proteins that regulate aerobic glycolysis are expressed at high levels in BL cells

**Fig 3:**
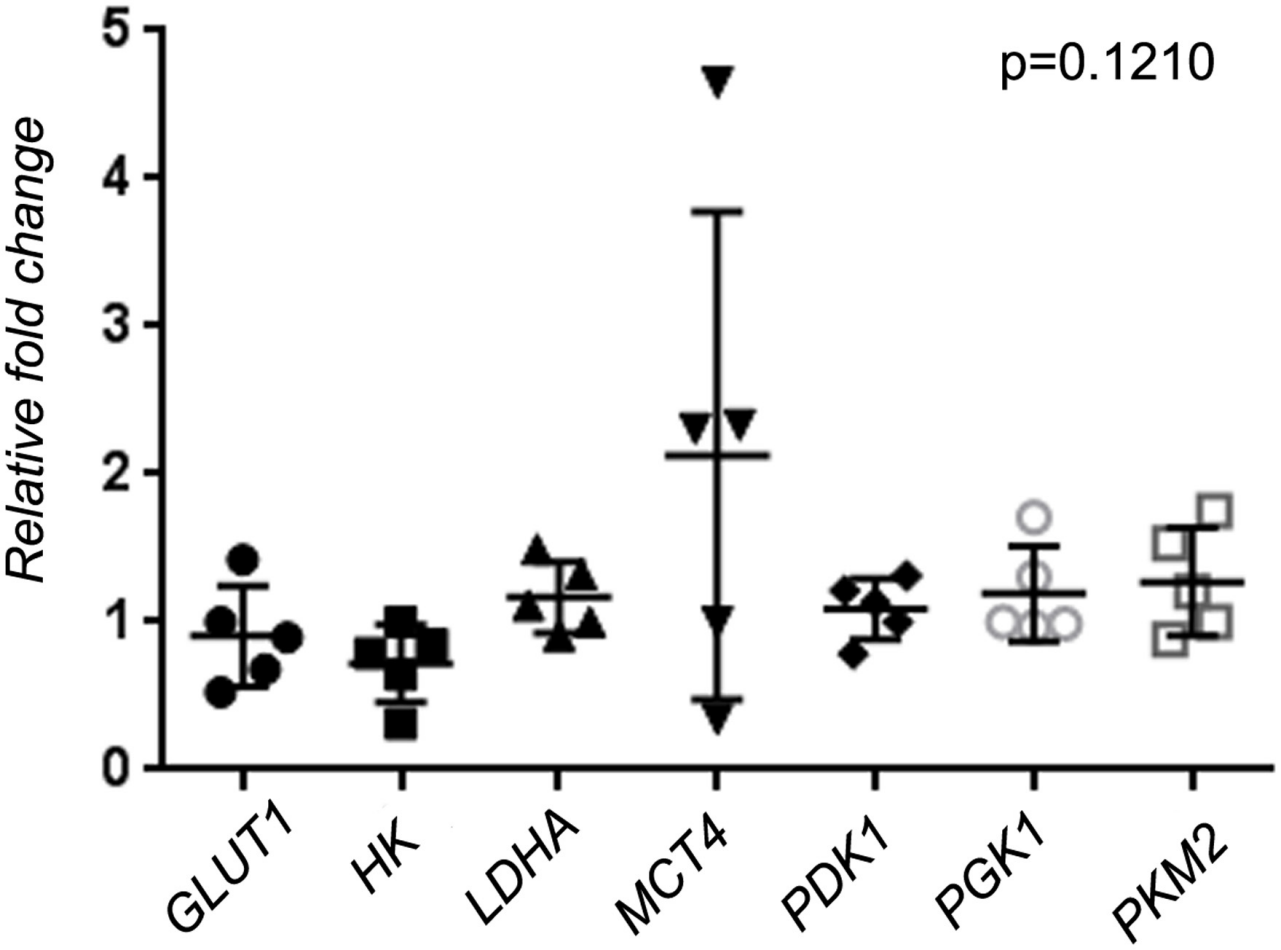
Expression levels of genes involved in the glycolytic pathway. We studied isogenic BL41 (Bl41, EBV negative; BL41/B95.8, Latency III) and Mutu (Mutu, EBV negative; Mutu I, Latency I; and Mutu III, Latency III) BL cell lines, and LCL121028 cells (5 months old). No significant differences in expression levels were observed in the five groups of cells for the studied genes. Each point represents the median value for three Q-PCR reactions. No value differed by more than 30% of the means.

The expression levels of HIF1A-responsive genes were measured quantitatively in the different BL cell lines, both EBV-positive and-negative (Fig 3). Two groups of isogenic cells were tested; namely, BL41 (EBV negative) and BL41/B-95.8 (Latency III), and also Mutu (cl. 30, EBV negative), Mutu I (cl. 148, Latency I), and Mutu III (cl. 176, Latency III). Levels of expression of *GLUTI*, *LDHA*, *MCT4*, *PDK1*, *PGK1*, and *PKM2* genes were moderately high and slightly higher in Latency III cells, in comparison with Latency I and EBV-negative cells. However, this difference was not significant in Kruskal–Wallis tests (p = 0.1210>0.05). Each point on Fig 3 represents a median value for three Q-PCR reactions. Note that *MCT4* expression was very high in BL cell lines. This suggests the enhanced production of lactate and its export from the cells.

### Biochemical characteristics of BL cell lines

**Fig 4:**
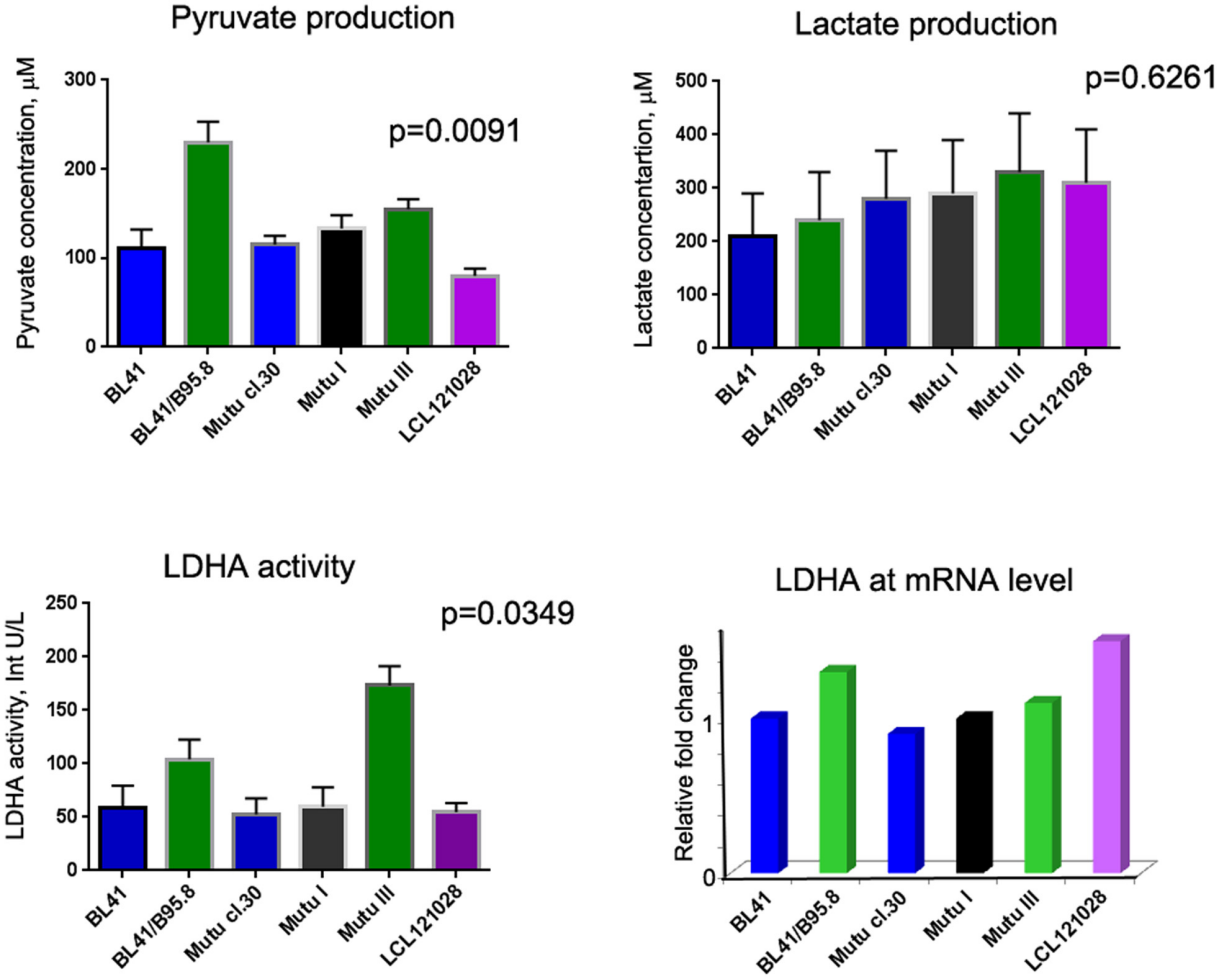
Biochemical characteristics of BL cell lines. The BL cells described in Fig 3 and LCL121028 cells were assayed for the concentrations of l-lactate and pyruvate, and for lactate dehydrogenase (LDHA) catalytic activity by colorimetric assays (see Materials and methods section). Latency III BL cells showed significantly higher levels of pyruvate production and LDHA activity (p = 0.0091 and p = 0.0349, respectively). There were no differences in LDHA expression at mRNA levels (no value differed more than 30% from the means). Lactate concentrations were similar in all five studied groups.

The concentrations of lactate and pyruvate were measured by spectrophotometry (Table 1). The concentration of pyruvate was higher in BL lines than in LCLs (Fig 4). This suggests that BLs might proliferate faster than LCLs. All studied cell lines produced similarly high levels of lactate. There were no dramatic differences in the lactate dehydrogenase (LDHA) expression levels at the mRNA level (Fig 4). However, the LDHA signal was slightly higher in Latency III BL cells and LCLs, and the catalytic activity of LDHA increased significantly in the Latency III BL cell lines (Fig 4). This could be explained by partial HIF1A protein stabilization effected by the EBNA-3 and EBNA-5 expressed by these cells.

**Table 1:** Biochemical characteristics of B-cell lines

Cell lines	EBV status, latency	Concentration of Pyruvate, μM	Concentration of Lactate, μM	LDHA activity, IntU/L
BL41	EBV-	111±21	210±80	58,4±21,1
BL41/95	EBV+, latency I	230±23	240±90	100,95±23,0
MUTUI	EBV+, latency I	115±10	280±90	52,59±15,1
MUTUIII	EBV+, latency III	155±11	330±110	173,17±18,2
LCL	EBV+, latency III	80±8	310±100	54,78±8,4

### An inhibitory effect of acriflavine hydrochloride on expression pattern of HIF1A responsive genes in LCLs

**Fig 5:**
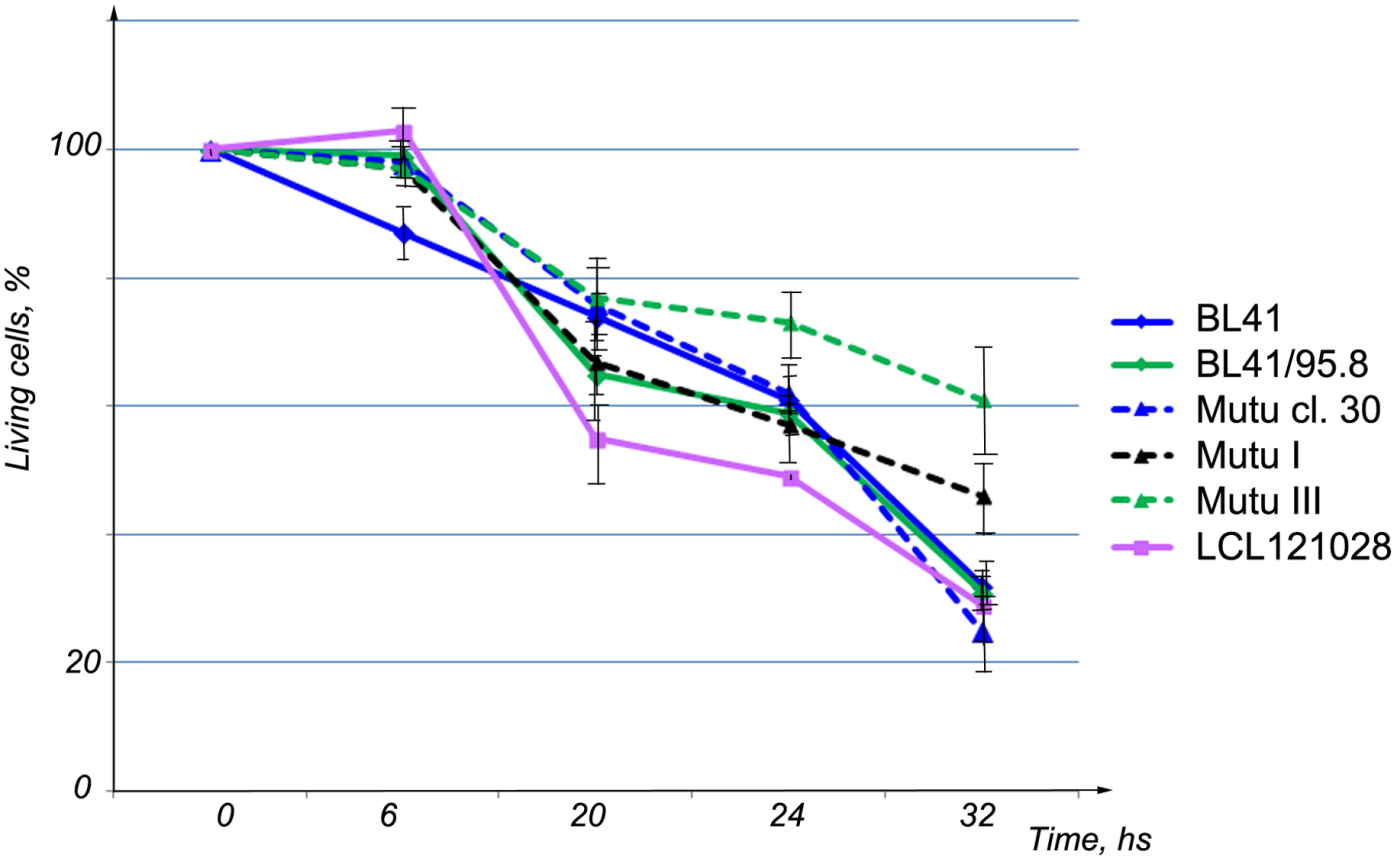
Influence of ACF on the proliferation of BL cell lines and LCLs. The percentage of living cells is presented as a function of the time of treatment. LCL121028 cells were quickly killed by ACF.

To test whether HIF1A might be responsible for the transcriptional activation of genes involved in regulation of aerobic glycolysis in BL cells of Latency III, the transactivating ability of HIF1A was inhibited. It is known that this transcription factor is active only as a heterodimer with ARNT [18]. Hence, formation of a protein complex between HIF1A and ARNT was prevented, using acriflavine hydrochloride (ACF) to inhibit this binding [19]. To test cell viability, cells were grown in medium containing 5 μM ACF. Cells were collected at 1–48 hours, and the numbers of living cells were counted. Massive cell death was observed after 20 h (Fig 5). To avoid any influence of cell death on Q-PCR results, probes were collected after 3 h of treatment with ACF.

**Fig 6:**
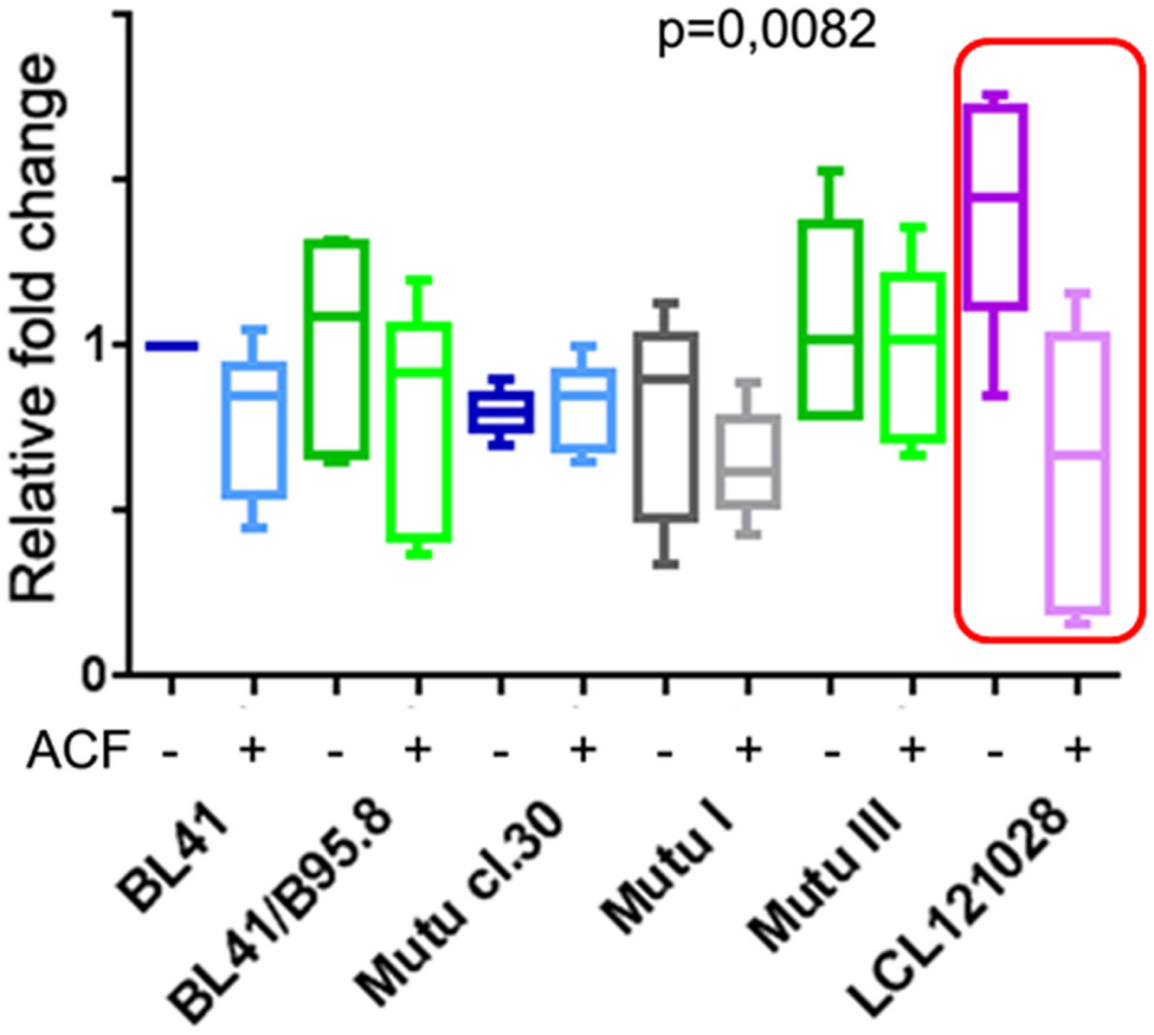
Expression levels of genes involved in glycolysis upon treatment with ACF. Expression levels of a set of genes (*GLUTI*, *HK*, *LDHA*, *MCT4*, *PDK1*, *PGK1*, and *PKM2*) were assessed by Q-PCR after the treatment of cells with 5 μM of ACF for 3 h. Kruskal–Wallis tests were applied to the results for 12 groups; namely, two EBV-negative, one Latency I, two Latency III BL cell lines, and LCL121028 cells—controls and treated with ACF. Note the significant decrease in gene expression under ACF treatment in LCLs (p = 0.0082; framed in the Fig). The median value for three Q-PCR reactions is shown; the standard deviation did not exceed 30% of the means.

All the tested HIF1A-dependent genes (*GLUTI*, *HK*, *LDHA*, *MCT4*, *PDK1*, *PGK1*, and *PKM2*) were significantly downregulated in LCLs after treatment with ACF (p<0.05, Fig 6). This suggests that ACF could inhibit the transactivating ability of HIF1A. However, there were no statistically significant changes in the expression levels of the same genes in different BL cell lines (Fig 6).

It was reported earlier [20] that 2-methoxyestradiol (2-MeOE2) inhibited nuclear translocation of HIF1A and HIF1B, and, as consequence, transcription of the HIF1A-dependent genes decreased. Expression of a set of genes (*GLUTI*, *HK*, *LDHA*, *PDK1*, *PGK1*, and *PKM2*) was assessed in BL cell lines and LCL121028 upon the treatment with 5 μM of 2-MeOE2 (see S2 Table). And again, expression of the abovementioned genes was decreased significantly (p = 0.0001) only in LCLs, but not in BL cell lines.

Thus, HIF1A played a limited role in the transactivation of genes involved in aerobic glycolysis in BL cell lines, in contrast to LCLs.

### Inhibition of MYC transactivating function results in decreased glycolysis in BL cells but not in LCLs

As mentioned above, BLs are characterized by an upregulated expression of *MYC*, arising from chromosomal rearrangements [21]. In addition, MYC induces a set of genes involved in glycolysis [11]. Hence, our next question was whether inhibition of the transactivating ability of MYC would result in the downregulation of those genes.

**Fig 7:**
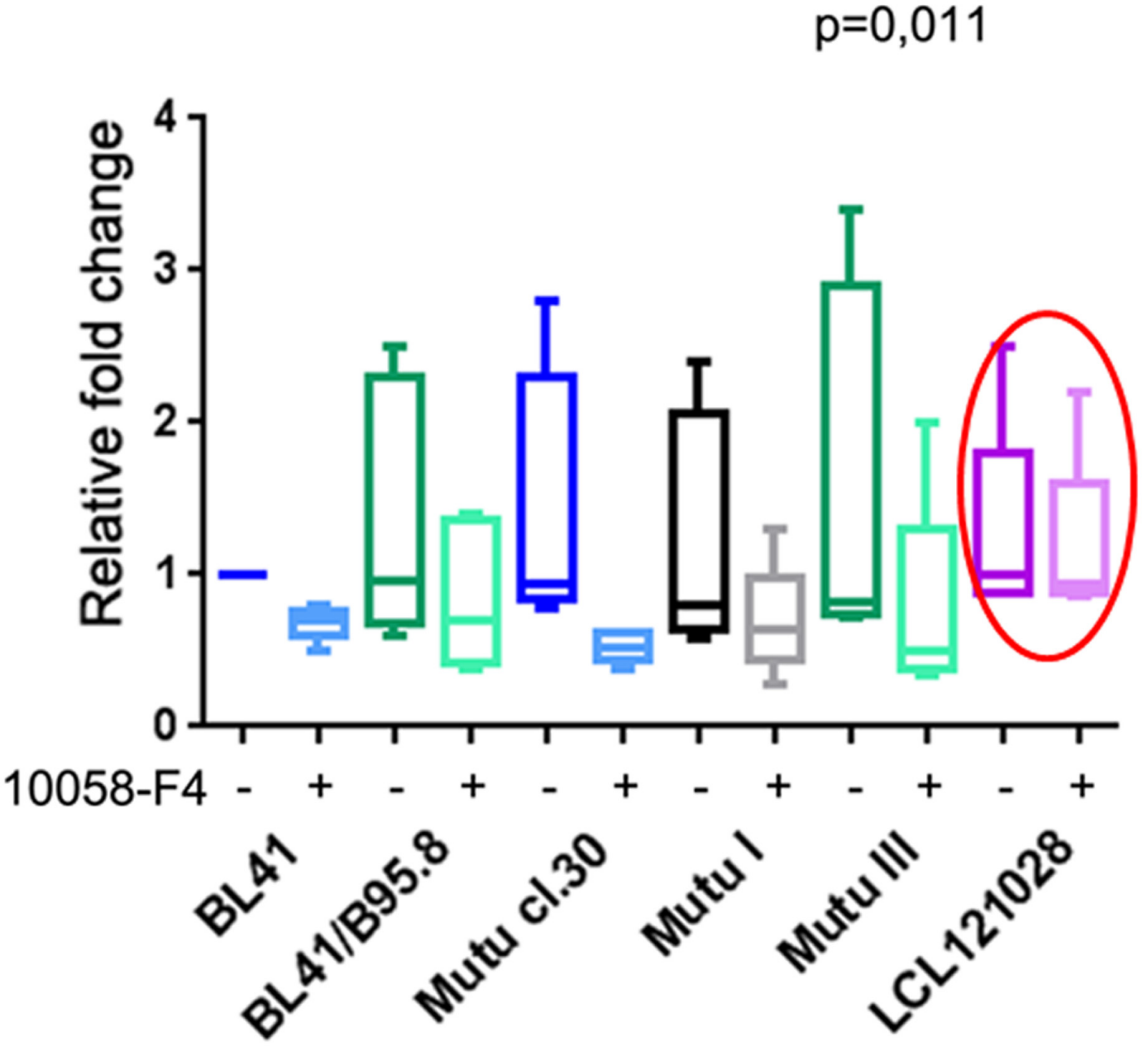
Expression levels of genes involved in glycolysis upon treatment with 10058-F4. Expression levels of a set of genes (*GLUTI*, *HK*, *LDHA*, *MCT4*, *PDK1*, *PGK1*, and *PKM2*) were assessed by Q-PCR after the treatment of cells with 100 μM of 10058-F4 for 4 h. Kruskal–Wallis tests were applied to the results for 12 groups: two EBV-negative, one Latency I, and two Latency III BL cell lines, and LCL121028 cells—controls and treated with 10058-F4. Note the significant decreases in gene expression levels under 10058-F4 treatment in the BL cell lines (p = 0.011), in contrast with no change in LCL121028 cells (encircled). The median value for three Q-PCR reactions is shown; the standard deviation did not exceed 30% of the means.

The agent 10058-F4 (5-[(4-ethylphenyl)methylene]-2-thioxo-4-thiazolidinone) was used to abolish the transactivating ability of MYC. This prevents dimerization of the MYC and MAX proteins, thus inhibiting the transactivation of MYC-dependent genes [22]. Cell growth medium was supplemented with 100 μM of 10058-F4. Cells were treated for 4 h, and the expression levels of genes involved in glycolysis (*GLUTI*, *HK*, *LDHA*, *MCT4*, *PDK1*, *PGK1*, and *PKM2*) were assessed by Q-PCR. Genes responsible for the Warburg effect were downregulated in all the BLs regardless of the presence or absence of EBV and the type of latency (Fig 7). Importantly, no significant decreases in gene expression levels were observed in LCLs in the same conditions (Fig 7). Similar results were obtained when 10074-G5, another MYC-inhibitor (described in [23]) was used (S2 Table). This suggests that MYC functions as a transcription factor to activate genes involved in aerobic glycolysis in BL cells but not in LCLs. In other words, HIF1A is the main regulator of the Warburg effect in LCLs, consistent with our previous data [10].

**Fig 8:**
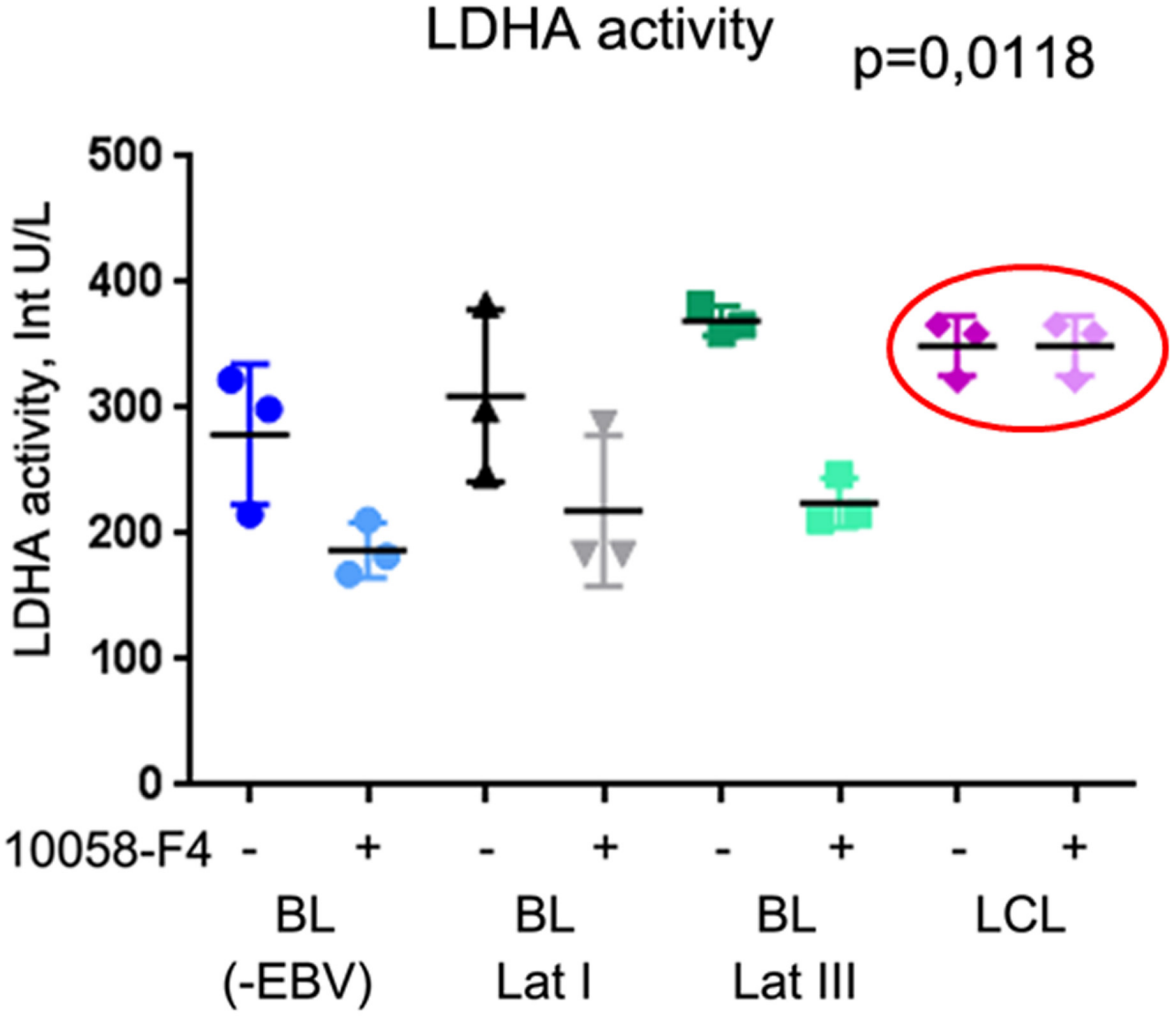
Lactate dehydrogenase activity in BL cell lines and LCLs. LDHA catalytic activity was measured in the control cells and after treatment with 100 μM of 10058-F4 for 4 h. The median value of three measurements (standard deviation not exceeding 30%) is shown on the Fig. Each of the four groups consisted of three cell lines: EBV-negative (Ramos, Bl28, DG75), Latency I (BL28/B95.8, Rael, Akata (+)), and Latency III (BL16, BL18, RAJI) BL cell lines, and three LCLs (051128, 111210, and 120214). Kruskal–Wallis tests were applied to the results of eight groups (controls and those treated with 10058-F4). Note the significant decrease of LDHA activity in all the BL lines upon inhibition of MYC transactivation ability (p = 0.0118). The catalytic activity of LDHA was not changed in LCLs (encircled).

Next, the LDHA activity was assayed in a set of BL cell lines and LCLs, under normal conditions and with 10058-F4 treatment. Four groups of cells were studied; namely, EBV-negative BL cell lines (Ramos, Bl28, DG75), Latency I (BL28/B95.8, Rael, Akata (+)), and Latency III (BL16, BL18, RAJI), and three LCLs (051128, 111210, and 120214). The LDHA activity was decreased in all the BLs upon MYC inhibition but not in the LCLs (Fig 8). This suggests that aerobic glycolysis is controlled mainly by MYC in BL cell lines, in contrast to HIF1A in LCLs.

## Discussion

As mentioned above, the Warburg effect is one of the hallmarks of cancer cells that can also be observed in rapidly proliferating cells such as activated T-cells [24]. We have shown previously that LCLs can also exhibit the Warburg effect; thus, lymphoblastoid cells show features that are characteristic for malignant and also for rapidly proliferating cells [10]. We have also shown that stabilized HIF1A transactivated those genes involved in glycolysis. Stabilization of the HIF1A protein was caused by the lack of hydroxylation from inactivation of prolylhydroxylases PHD1 and PHD2. The catalytic activity of PHD1 and PHD2 is inhibited because they form protein complexes with the EBV-encoded nuclear antigens EBNA-5 and EBNA-3, respectively.

The molecular regulation of aerobic glycolysis in BL cells is not yet fully understood. Using the model BL cell line P493-6 that carries an inducible *MYC* repression system, it was suggested that at least two genes encoding enzymes for glycolysis (*HK2* and *PDK1*) are regulated by the HIF1A and MYC proteins cooperatively [25].

To elucidate glycolytic pathway regulation in BLs, we studied the expression patterns of *GLUTI*, *HK*, *LDHA*, *MCT4*, *PDK1*, *PGK1*, and *PKM2* encoding enzymes for glycolytic pathway. All these genes were expressed at high levels in BL lines and LCLs (Fig 3). Therefore, what is the main regulator of expression in BL lines?

We found that the HIF1A protein was expressed at high levels in EBV-positive BLs, especially in Latency III cells (Figs 1 and 2). To monitor the importance of HIF1A function in BL cells, the transcriptional activity of HIF1A was inhibited by ACF to retain HIF1A in the cytoplasm, thus preventing HIF1A–ARNT heterodimer formation [19]. HIF1A-induced genes, such as *GLUT1*, *HK*, *PGK1*, *PDK1*, *MCT4*, *PKM2*, and *LDHA* were downregulated significantly in LCLs, in contrast to BL cells that showed no such inhibition (Fig 6). It is known that treatment with ACF might lead to the inhibition of lipopolysaccharide-induced NF-κB activation [26]. Notably, ACF can induce apoptosis and necrosis in yeast cells [27]; therefore, cells were treated with ACF for only 3 h when massive cell death had not yet occurred (Fig 5). The results suggest that HIF1A is not a major factor in the regulation of aerobic glycolysis in BLs.

As discussed above, MYC is another transcription factor activating the transcription of genes involved in glycolysis. However, its expression is quite low in LCLs, even though the EBNA-2 protein activates the *MYC* promoter through interactions with the CBP (cAMP-response element (CREB)-binding protein, NP_001073315) and histone acetyl transferase P/CAF (p300/CBP-associated factor; NP_003875) [28]. In BL cells, MYC is expressed at high levels because of gene activation by chromosome translocation. To monitor the consequences of inhibition of MYC transactivating ability, LCLs and BLs were treated with 10058-F4 to prevent the binding of MYC and Max proteins. No significant changes in responsive gene expression levels were detected in the treated LCLs, compared with controls, in contrast with BL cell lines (Fig 7). Moreover, the control and treated LCLs, unlike BL cells, showed the same high activity of LDHA that converts pyruvate into lactate during aerobic glycolysis (Fig 8). The expression levels of the same set of genes (*HK*, *MCT4*, *PDK1*, *HK*, and *LDHA*) were downregulated upon inhibition of MYC transcriptional activity (Fig 7). The LDHA activity was significantly decreased in BLs treated with 10058-F4, compared with the control BL cells (Fig 8).

In conclusion, we suggest that aerobic glycolysis—the Warburg effect—in BL cells is regulated by high levels of MYC, unlike LCLs where HIF1A is responsible for this phenomenon.

## Supporting Information

S1 Table Latency of BL cell lines used in experiments. (DOCX)

S2 Table Expression levels of genes involved in glycolysis upon treatment with 2-methoxyestradiol (2-MeOE2) and 10074-G5, assessed by Q-PCR, presented as the relative fold change. (DOCX)
