# Supplementary material for: Different Mechanisms of Regulation of the Warburg Effect in Lymphoblastoid and Burkitt Lymphoma Cells
Source: PLoS One. 2015 Aug 27;10(8):e0136142. doi: 10.1371/journal.pone.0136142 (PMC4551852; doi:10.1371/journal.pone.0136142)
Supplement: S1 Table — (DOCX) [file pone.0136142.s001.docx]

**S1Table.** Latency of BL cell lines used in experiments

| **Cells** | **EBV status, latency** |
| --- | --- |
|  |  |
| DG75 | EBV negative |
| Ramos | EBV negative |
| Rael | latency I |
| Akuba | latency III |
| BL16 | latency III |
| BL18 | latency III |
| RAJI | latency III |
| **Isogenic cells** | |
| Akata | EBV negative |
| Akata (+) | latency I |
| BL28 | EBV negative |
| BL28/B95.8 | latency I |
| BL41 | EBV negative |
| BL41/B95.8 | latency III |
| BJIAB | EBV negative |
| BJIAB/B95.8 | latency I |
| BJAB-EBNA-1 | BJAB that expressed EBNA-1 constitutively |
| Jijoye M13 | latency I |
| Jijoye P79 | latency III |
| Oma clone 4 | EBV negative |
| Oma clone 6 | latency I |
| Mutu clones 9 and 30 | EBV negative |
| Mutu I clones 59 and 148 | latency I |
| Mutu III clones 99 and 176 | latency III |
