# Supplementary material for: Different Mechanisms of Regulation of the Warburg Effect in Lymphoblastoid and Burkitt Lymphoma Cells
Source: PLoS One. 2015 Aug 27;10(8):e0136142. doi: 10.1371/journal.pone.0136142 (PMC4551852; doi:10.1371/journal.pone.0136142)
Supplement: S2 Table — (DOCX) [file pone.0136142.s002.docx]

**S2 Table.** Expression levels of genes involved in glycolysis upon treatment with 2-methoxyestradiol (2-MeOE2) and 10074-G5, assessed by Q-PCR, presented as relative fold change.

| Gene↓/Cells→ | Mutu clone 30 | Mutu clone 30 | Mutu I clone 148 | Mutu Iclone 148 | Mutu III clone 99 | Mutu III clone 99 | LCL  121028 | LCL  121028 |
| --- | --- | --- | --- | --- | --- | --- | --- | --- |
| **2-MeOE2** | **-** | **+** | **-** | **+** | **-** | **+** | **-** | **+** |
| *GLUT1* | 1 | 0,88 | 0,8 | 0,82 | 0,9 | 1,04 | 1,2 | 0,5 |
| *HK* | 1 | 0,9 | 0,7 | 0,8 | 0,85 | 0,91 | 1,15 | 0,7 |
| *LDHA* | 1 | 0,8 | 0,91 | 0,57 | 1,14 | 1 | 1,49 | 0,73 |
| *PDK1* | 1 | 0,9 | 1,13 | 0,9 | 0,95 | 1,15 | 1,21 | 0,72 |
| *PGK1* | 1 | 0,95 | 1 | 0,7 | 1,35 | 1,2 | 1,8 | 1,2 |
| *PKM2* | 1 | 0,88 | 0,9 | 0,8 | 1,6 | 1,4 | 1,6 | 0,95 |
| **10074-G5** | **-** | **+** | **-** | **+** | **-** | **+** | **-** | **+** |
| *GLUT1* | 1 | 0,65 | 0,9 | 0,7 | 0,74 | 0,5 | 0,91 | 0,9 |
| *HK* | 1 | 0,52 | 0,8 | 0,56 | 0,83 | 0,6 | 0,87 | 0,85 |
| *LDHA* | 1 | 0,5 | 0,7 | 0,52 | 0,72 | 0,39 | 1 | 0,94 |
| *PDK1* | 1 | 0,42 | 0,9 | 0,45 | 1,1 | 0,72 | 1,2 | 1,3 |
| *PGK1* | 1 | 0,6 | 0,8 | 0,62 | 1,1 | 0,8 | 1,2 | 1,05 |
| *PKM2* | 1 | 0,75 | 0,8 | 0,5 | 0,9 | 0,6 | 1,1 | 0,99 |

Expression levels of a set of genes (GLUTI, HK, LDHA, PDK1, PGK1, and PKM2) were assessed by Q-PCR after the treatment of cells with 5 μM of 2-MeOE2 and 100 μM of 10074-G5 for 3 h. Kruskal–Wallis tests were applied to the results for 8 groups; namely, one EBV-negative (Mutu clone 30), one Latency I (Mutu I clone 148), and one Latency III (Mutu III clone 99) BL cell lines, and LCL121028 cells - controls and treated with abovementioned chemicals. Note the significant decrease in gene expression under 2-MeOE2 treatment in LCLs (p=0.0001) and under 10074-G5 treatment in BL cells (p<0.0001). The median value for three Q-PCR reactions is shown; the standard deviation did not exceed 30% of the means.
